# Supplementary material for: Analysis of PPARγ Signaling Activity in Psoriasis
Source: Int J Mol Sci. 2021 Aug 10;22(16):8603. doi: 10.3390/ijms22168603 (PMC8395241; doi:10.3390/ijms22168603)
Supplement: Supplementary file 1 [file ijms-22-08603-s001.zip › Supplemental materials_Analysis of PPARg signaling activity in psoriasis/Pathway models/Models images and html files/Interleukin-17 and interleukin-22 signaling in psoriasis/10426.html]

MAP2K4


# Protein MAP2K4

|  |  |
| --- | --- |
| URN | urn:agi-llid:6416 |
| Total Entities | 0 |
| Connectivity | 1225 |
| Name | MAP2K4 |
| Description | mitogen-activated protein kinase kinase 4 |
| Notes | This gene encodes a member of the mitogen-activated protein kinase (MAPK) family. Members of this family act as an integration point for multiple biochemical signals and are involved in a wide variety of cellular processes such as proliferation, differentiation, transcription regulation, and development. They form a three-tiered signaling module composed of MAPKKKs, MAPKKs, and MAPKs. This protein is phosphorylated at serine and threonine residues by MAPKKKs and subsequently phosphorylates downstream MAPK targets at threonine and tyrosine residues. A similar protein in mouse has been reported to play a role in liver organogenesis. A pseudogene of this gene is located on the long arm of chromosome X. Alternative splicing results in multiple transcript variants. [provided by RefSeq, Jul 2013] |
| Primary Cell Localization | Cytoplasm |
| Class | Protein kinase |
| ObjectType | Protein |

---

|  |  |
| --- | --- |
| Pathway | Osteoarthritis |
|  | Skin Aging |
|  | MTHFR Activity Regulation |
|  | Dopamine Receptors Signaling |
|  | Thrombopoietin Receptors Signaling in Platelet Maturation |
|  | Toll-like Receptors Act through MYD88-TIRAP Signaling |
|  | TLR4 Signaling in Leukocytes |
|  | Plasmin Effects in Inflammation |
|  | Scavenger Receptors in Platelet Activation |
|  | Toll-like Receptors in Sterile Inflammation |
|  | Basophil Activation |
|  | Eosinophil Survival by Cytokine Signaling |
|  | AXL Receptor Inhibits Macrophages and Dendritic Cells Function |
|  | Neutrophil Recruitment and Priming |
|  | Negative Acute Phase Proteins Synthesis |
|  | Positive Acute Phase Proteins Synthesis |
|  | Vascular Endothelial Cell Activation by Cytokines |
|  | Vascular Endothelial Cell Activation by Growth Factors |
|  | Nociception Expression Targets Signaling |
|  | EDNRA/B -> Vascular Motility |
|  | ADRA1A -> IL6 Production |
|  | CNR1/2 -> IL1B/2/4/6/10 Production |
|  | DRD2 Expression Targets |
|  | PTGER2/3 -> Inflammation-Related Expression Targets |
|  | Circadian Clock Genes in Suprachiasmatic Nuclei Neurons |
|  | Cardiomyocyte Dysfunction |
|  | Scavenger Receptor OLR1 in Inflammation Related Endothelial Dysfunction |
|  | Dendritic Cells Function in Atherosclerosis |
|  | Dilated Cardiomyopathy |
|  | Myocardial Remodeling |
|  | Myocardial Infarction |
|  | Inflammatory Reaction in Acne Vulgaris |
|  | Mast-Cells Activation in Atopic Dermatitis |
|  | IL17 Signaling in Psoriasis |
|  | Dendritic Cells Function in Psoriasis |
|  | Mesangial Cell Dysfunction in Diabetic Nephropathy |
|  | Insulin Resistance in Myocytes Induced by Obesity |
|  | Insulin Resistance in Hepatocytes |
|  | beta-Cell Destruction through Cytokines in Diabetes Mellitus |
|  | GNRH Signaling Impairment in Hypogonadotrophic Hypogonadism |
|  | OipA Signaling in Helicobacter Pylori Infection |
|  | Dendritic Cell Function in Ulcerative Colitis |
|  | Antiphospholipid Antibodies in Endothelial Cell |
|  | B-Cell Acute Lymphoblastic Leukemia |
|  | B-Cell Chronic Lymphocytic Leukemia |
|  | Chronic Myeloid Leukemia |
|  | Follicular Lymphoma |
|  | Osteoclast Activation in Multiple Myeloma |
|  | Multiple Myeloma |
|  | Antiphospholipid Antibodies and Endothelial Cell Interaction |
|  | T-Cell Acute Lymphoblastic Leukemia |
|  | Hepatocellular Carcinoma |
|  | Growth Factor Signaling in Hepatocellular Carcinoma |
|  | Proteins Involved in Pancreatic Cancer |
|  | Pancreatic Ductal Carcinoma |
|  | Growth Factor Signaling in Pancreatic Neoplasms |
|  | Mucin Production in Goblet Airway Epithelial Cells |
|  | CFTR Facilitates Oxidative Stress in Airway Epithelium in CF |
|  | Airway Epithelial Cell Dysfunction |
|  | Osteoclast Activation in Gout |
|  | Neutrophil Recruitment in Sinovium in Gout |
|  | Monocytes Function in Gout |
|  | TNF and IL1B Induce Metalloproteinase Synthesis in Osteoarthritis |
|  | Dendritic Cell Dysfunction in Psoriatic Arthritis |
|  | Synovial Fibroblast Activation in Psoriatic Arthritis |
|  | Keratinocyte Activation in Psoriatic Arthritis |
|  | Osteoclast Activation in Rheumatoid Arthritis |
|  | Synovial Fibroblast Activation by Citokines in Rheumatoid Arthritis |
|  | TLR2 Induced Synovial Fibroblast Activation in Rheumatoid Arthritis |
|  | Autocrine Cytokine/Chemokine Loops in Systemic Scleroderma |
|  | Skin Fibrosis |
|  | CCR5 Signaling in Macrophages |
|  | Majeed Syndrome |
|  | IL6 Promotes Inflammation in Muscular Dystrophies |
|  | Osteoclast Activation in Postmenopause |
|  | Glioma Invasion Signaling |
|  | Growth Factor Signaling in Neuroblastoma |
|  | Neuroblastoma |
|  | WNT Signaling in Breast Cancer |
|  | ESR1/ERBB Positive Luminal Breast Cancer |
|  | ESR1 Signaling in Breast Cancer |
|  | Endometrial Cancer |
|  | Endometrioid Endometrial Cancer |
|  | Proteins Involved in Endometrial Cancer |
|  | Clear Cell Endometrial Cancer and Papillary Serous Endometrial Cancer |
|  | Angiogenesis in Endometriosis |
|  | Proteins Involved in High-Grade Serous Ovarian Carcinoma |
|  | Endometrioid Ovarian Carcinoma |
|  | Proteins Involved in Endometrioid Ovarian Carcinoma |
|  | Clear Cell Ovarian Carcinoma |
|  | Proteins Involved in Mucinous Ovarian Carcinoma |
|  | High-Grade Serous Ovarian Carcinoma |
|  | Low-Grade Serous Ovarian Carcinoma |
|  | Proteins Involved in Low-Grade Serous Ovarian Carcinoma |
|  | Ovarian Cancer |
|  | Proteins Involved in Clear Cell Ovarian Carcinoma |
|  | Mucinous Ovarian Carcinoma |
|  | Subepithelial Fibroblasts in Middle Ear Cholesteatoma |
|  | HPV E5 Regulates EDNRA and EGFR Signaling Pathway in Keratinocytes |
|  | Androgen Receptor/Akt Signaling |
|  | Steroids Induced Cataract |
|  | VEGFA in Neovascular Glaucoma |
|  | Cytokines Trigger Otitis Media |
|  | Immune System Role in Otitis Media |
|  | Alveolar Macrophages Dysfunction in COPD |
|  | T-Cell Cytotoxic Activation |
|  | Mucin Hyperproduction in Goblet and Mucous Cells |
|  | WNT Signaling in Cystic Kidney Disease |
|  | Polycystins Mutation Effects in Cystic Kidney Disease |
|  | Plasmin Role in Glomerulonephritis |
|  | Endothelial Cell Dysfunction in Glomerulonephritis |
|  | Mesangial Cell Dysfunction in Glomerulonephritis |
|  | Interstitial Fibroblasts in Pyelonephritis |
|  | Apoptosis of Renal Tubule Epithelial Cell in Pyelonephritis |
|  | Endothelial Cell Dysfunction in Pyelonephritis |
|  | ANGPT1 -> CREB/CREBBP Expression Targets |
|  | ANGPT2 -> AP-1/CREBBP/MYC Expression Targets |
|  | AREG -> CREB/CREBBP Expression Targets |
|  | AREG -> AP-1 Expression Targets |
|  | Dopamine/Gi Expression Targets |
|  | BTC -> AP-1/ATF/CREB Expression Targets |
|  | Frizzled Receptors -> ARRB1/ARRB2 non-Canonical Signaling |
|  | AGTR1 -> ARRB1/ARRB2 Signaling |
|  | Sialophorin -> CTNNB/MYC/TP53 Signaling |
|  | CD72 -> NF-kB Expression Targets |
|  | CD8 -> NF-kB Expression Targets |
|  | CD80 -> NFATC Expression Targets |
|  | CD86 -> NFATC Expression Targets |
|  | EGF -> AP-1/ATF Expression Targets |
|  | EGF -> CREB/CREBBP/ELK/SRF/MYC Expression Targets |
|  | EGF -> TP53 Expression Targets |
|  | EGFR/ERBB2 -> TP53 Signaling |
|  | EGFR -> AP-1/ATF2 Signaling |
|  | EGFR -> AP-1/CREB/ELK/SRF/MYC Signaling |
|  | EREG -> CREB Expression Target |
|  | EREG -> AP-1/ATF Expression Targets |
|  | Erythropoietin -> AP-1/MYC/CREB Expression Targets |
|  | F2 -> AP-1/CREB/ELK/SRF/SP1 Expression Targets |
|  | PLG -> AP-1/CREB/ELK/SRF/SP1 Expression Targets |
|  | FGF9 -> AP-1/CREB/MYC Expression Targets |
|  | FGF10 -> AP-1/CREB/CREBBP/MYC Expression Targets |
|  | FGF1 -> AP-1/CREB/ELK/SRF/MYC Expression Targets |
|  | FGF8 -> AP-1/CREB/MYC Expression Targets |
|  | FGF18 -> AP-1/CREB Expression Targets |
|  | FGF4 -> AP-1/MYC Expression Targets |
|  | FGF7 -> AP-1/CREB/CREBBP/MYC Expression Targets |
|  | FGF2 -> AP-1/CREB/CREBBP/ELK/SRF/MYC Expression Targets |
|  | FGFR -> AP-1/CREB/CREBBP/ELK/SRF/MYC Signaling |
|  | FrizzledR -> JUN/PAX2 Signaling |
|  | HBEGF -> AP-1/ATF Expression Targets |
|  | HBEGF -> TP53 Expression Targets |
|  | HBEGF -> CREB/MYC Expression Targets |
|  | HGF -> AP-1/CREB/ELK/SRF/MYC Expression Targets |
|  | HGFR -> AP-1/CREB/MYC Signaling |
|  | RAS/RAF/MAPK Signaling Activation in Cancer |
|  | RAS/RAF/MAPK Signaling Activation by Blocking of Tumor Suppressors |
|  | TGFB Signaling activation by Blocking of Tumor Suppressors |
|  | Apoptosis Block by Death Receptor Signaling in Cancer |
|  | Apoptosis Evasion in Cancer: Overview |
|  | VEGFA Dependent Angiogenesis in Cancer |
|  | Inhibition of THBS1 Activates Angiogenesis in Cancer |
|  | VEGF Independent Angiogenesis in Cancer |
|  | TGFB Family in Epithelial to Mesenchymal Transition in Cancer |
|  | FibronectinR -> NF-kB Signaling |
|  | FibronectinR -> AP-1/ELK/SRF/SREBF Signaling |
|  | Fibronectin Expression Targets |
|  | CTGF -> AP-1/CREB/MYC Expression Targets |
|  | IL1R -> STAT3 Signaling |
|  | IL16-> ATF/CREB/CREBBP Expression Target |
|  | PAF/Gq -> AP-1/ATF1/CREB/ERK/SRF Expression Targets |
|  | Dronabinol/Anandamide Expression Targets |
|  | S1P Expression Targets |
|  | NRG1 -> CREB/CREBBP/ELK/SRF/MYC Expression Targets |
|  | NRG1 -> TP53 Expression Target |
|  | NRG1 -> AP-1/ATF Expression Targets |
|  | NGF -> SMAD3/NF-kB Expression Targets |
|  | NGF -> AP-1/TP53/MYC Expression Targets |
|  | BDNF Expression Targets |
|  | NTF3 Expression Targets |
|  | NTF4 Expression Targets |
|  | NGF -> CREB/CEBPB/MEF2A Expression Targets |
|  | NOTCH -> TCF3 Signaling |
|  | NOTCH Expression Targets |
|  | PDGFB -> AP-1/CREB/MYC Expression Target |
|  | PDGFC -> CREB Expression Target |
|  | PDGFD -> AP-1 Expression Targets |
|  | PDGF -> AP-1/CREB/CREBBP/MYC Expression Targets |
|  | PDGFR -> AP-1/MYC Signaling |
|  | AVP/Gs -> CREB/ELK/SRF/AP-1/EGR Expression Targets |
|  | GNRH1 Expression Targets |
|  | GNRH2 Expression Targets |
|  | EDN1 Expression Targets |
|  | EDN3 Expression Targets |
|  | AVP/Gq -> CREB/ELK/SRF/AP-1/EGR Expression Targets |
|  | PDCD1 -> ATF/CREB/CREBBP Expression Targets |
|  | AngiopoietinR -> AP-1 Signaling |
|  | GDNF -> HSF1 Signaling |
|  | EphrinB -> MAPK/JUN/FOS Signaling |
|  | NTRK -> AP-1/CREB/ELK/SRF/MYC/SMAD3/TP53 Signaling |
|  | GAS6 -> AP-1/CREB Expression Targets |
|  | FIGF -> AP-1 Expression Target |
|  | KITLG -> AP-1/CREB/CREBBP/MYC Expression Targets |
|  | GDNF -> HSF1 Expression Targets |
|  | CSF1 -> AP-1/CREB/CREBBP/MYC Expression Targets |
|  | PGF -> AP-1/CREB/CREBBP/MYC Expression Targets |
|  | FLT3LG -> AP-1/CREB/CREBBP Expression Targets |
|  | TGFBR -> AP-1 Signaling |
|  | TGFBR -> CREB/ELK-SRF Signaling |
|  | TGFBR -> ATF/GADD/MAX/TP53 Signaling |
|  | TGFBR -> MEF/MYOD/MYOG Signaling |
|  | TNF-alpha/TNFRSF1A Signaling |
|  | JNK/MAPK Signaling |
|  | TGF-beta Signaling |
|  | P38 MAPK/MAPK14 Signaling |
|  | AXL Receptor Tyrosine Kinase Signaling |
|  | PI3K/RAC1 Signaling |
|  | VEGF Signaling |
|  | EGFR Signaling |
|  | WNT Planar Cell Polarity (PCP) non-Canonical Signaling |
|  | TCR -> CREB/CREBBP/ATF Expression Targets |
|  | T-Cell Receptor -> ATF/CREB Signaling |
|  | TGFA -> TP53 Expression Targets |
|  | TGFA/AP-1/ATF Expression Targets |
|  | TGFA -> CREB/CREBBP/ELK-SRF/MYC Expression Targets |
|  | TGFB3-TGFBR2 Expression Targets |
|  | TGFB2-TGFBR2 Expression Targets |
|  | TGFB1-TGFBR2 Expression Targets |
|  | FASLG Expression Targets |
|  | TNFSF10 Expression Targets |
|  | LTA Expression Targets |
|  | TNFSF14 Expression Targets |
|  | TNF -> AP-1 Expression Targets |
|  | CD72 -> AP-1 Expression Targets |
|  | CD40LG -> NF-kB/ELK/SRF -> CREB/NFATC Expression Targets |
|  | TNFSF13 Expression Targets |
|  | TNFSF13B Expression Targets |
|  | TNF -> ELK-SRF Expression Targets |
|  | TNF -> CREB Expression Targets |
|  | TNF -> TP53/ATF Expression Targets |
|  | EDA Expression Targets |
|  | EctodysplasinR -> AP-1 Signaling |
|  | TNFR -> CREB/ELK-SRF Signaling |
|  | TNFR -> AP-1/ATF/TP53 Signaling |
|  | NGFR -> AP-1/CEBPB/CREB/ELK/SRF/TP53 Signaling |
|  | NGFR -> MEF Signaling |
|  | TNFRSF6 -> DDIT3 Signaling |
|  | TNFRSF6 -> HSF1 Signaling |
|  | TNFRSF1A -> AP-1/ATF/TP53 Signaling |
|  | TNFRSF1A -> CREB/ELK-SRF Signaling |
|  | Thrombopoietin -> SPI1 Expression Targets |
|  | Thrombopoietin -> AP-1/CREB/CREBBP/MYC Expression Targets |
|  | TLR4 -> AP-1 Expression Targets |
|  | TLR5 Expression Targets |
|  | TLR7 Expression Targets |
|  | TLR4 -> AP-1/EGR1/HIF1A Expression Targets |
|  | IL1B -> PGE2 Expression Targets |
|  | TLR1 -> 2/6 Expression Targets |
|  | IL1B Expression Targets |
|  | IL1A Expression Targets |
|  | TLR9 Expression Targets |
|  | TLR -> AP-1 Signaling |
|  | ROS in Angiotensin Mediated Cardiovascular Remodeling and Hypertrophy |
|  | VEGFA -> ATF/CREB/ELK-SRF Expression Targets |
|  | VEGFC -> ATF Expression Target |
|  | VEGFA -> AP-1/CREBBP/MYC Expression Targets |
|  | VEGFR -> AP-1/CREB/MYC Signaling |
|  | VEGFR -> ATF/CREB/ELK-SRF Signaling |
|  | WNT1 Expression Targets |
|  | WNT3A Expression Targets |
|  | WNT4 Expression Targets |
|  | WNT5A Expression Targets |
|  | WNT7A Expression Targets |
|  | 1\_Inflammation in keratinocytes in the pathology of acne |
|  | 2\_Acute phase initiation in atopic dermatitis |
|  | 2\_Interleukin-17 and interleukin-22 signaling in psoriasis |
|  | 1\_TNF and IL1B provoke ECM degradation in osteoarthritis |
|  | 1\_Osteoclast-mediated bone resorption in postmenopausal women |
|  | 1\_Formation of cysts in polycystic kidney disease |
|  | 2\_The role of endothelial cells in pyelonephritis |
|  | 3\_The role of interstitial fibroblasts in pyelonephritis |
|  | 3\_Mesangial cell dysfunction in glomerulonephritis |
|  | 1\_Disruption of luteinizing hormone and follicle-stimulating hormone secretion in PCOS |
|  | 2\_Pathogens stimulate mucins expression in the middle ear |
|  | 3\_Cardiomyocyte dysfunction unrelated to cell junctions in ARVD |
|  | 2\_Intestine epithelial cells drive immunological responses in ulcerative colitis |
|  | 6\_p12I Viral protein promotes T-cell activation in HTLV-1 infection |
|  | 4\_2\_Insulin resistance: inflammation-related insulin resistance |
|  | 4\_3\_Insulin resistance: FFA-related insulin resistance |
|  | 2\_Neuron and Schwann cell death |
|  | mapk1 protein modificators |
|  | Interleukin-17 and interleukin-22 signaling in psoriasis |

---

|  |  |
| --- | --- |
| Group | Metastasis Suppressors |

---

|  |  |
| --- | --- |
| MedScan ID | 6416 |

---

|  |  |
| --- | --- |
| LocusLink ID | 6416 |
|  | 26398 |
|  | 287398 |

---

|  |  |
| --- | --- |
| Alias | SAPK/ERK kinase I |
|  | Dual specificity mitogen-activated protein kinase kinase IV |
|  | SAPK kinase 1 |
|  | JNK-activated kinase 1 |
|  | SEK1/MKK4/JNKK1/SKK1 |
|  | JNKK1 |
|  | MKK4 |
|  | MAP2K4delta |
|  | MAPKK 4 |
|  | SEK1 |
|  | SAPK kinase I |
|  | SAPK/ERK kinase 1 |
|  | JNK kinase 1 |
|  | RP23-340L19.1 |
|  | MAPK kinase IV |
|  | SKK1/MKK4 |
|  | mitogen activated protein kinase kinase 4 |
|  | MAPK/ERK kinase IV |
|  | SAPKK-1 |
|  | c-Jun N- terminal kinase kinase 1 |
|  | stress activated protein kinase kinase 1 |
|  | SERK1 |
|  | c-Jun N- terminal kinase kinase I |
|  | MAPK kinase 4 |
|  | MEK4 |
|  | Dual specificity mitogen-activated protein kinase kinase 4 |
|  | JNKK I |
|  | mitogen activated protein kinase kinase 4, variant 1 |
|  | OTTMUSP00000006102 |
|  | SERK I |
|  | JNK activating kinase 1 |
|  | MAPK/ERK kinase 4 |
|  | JNK-activated kinase I |
|  | SKK1 |
|  | PRKMK4 |
|  | MAP kinase kinase 4 |
|  | MAP2K4 |
|  | mitogen-activated protein kinase kinase IV |
|  | MAP kinase kinase IV |
|  | SEK I |
|  | JNK activating kinase I |
|  | JNKK |
|  | MAPKK4 |
|  | SAPKK1 |
|  | JNK-activating kinase 1 |
|  | MEK 4 |
|  | c-Jun N-terminal kinase kinase 1 |
|  | stress-activated protein kinase kinase 1 |
|  | SAPK/Erk/kinase 1 |
|  | JNKK 1 |
|  | JNK kinase |
|  | mitogen-activated protein kinase kinase 4 |
|  | PRKMK4s |
|  | MAPKK4s |

---

|  |  |
| --- | --- |
| EC Number | 2.7.12.2 |
|  | 2.7.1.- |

---

|  |  |
| --- | --- |
| GO ID | 0005524 |
|  | 0008545 |
|  | 0004708 |
|  | 0031435 |
|  | 0004672 |
|  | 0004674 |
|  | 0004713 |
|  | 0038095 |
|  | 0007254 |
|  | 0007257 |
|  | 0000187 |
|  | 0032147 |
|  | 0006915 |
|  | 0061049 |
|  | 0071260 |
|  | 0072709 |
|  | 2000672 |
|  | 0018108 |
|  | 0045740 |
|  | 0043525 |
|  | 0051770 |
|  | 0034393 |
|  | 0009611 |
|  | 0007165 |
|  | 0023014 |
|  | 0031098 |
|  | 0030424 |
|  | 0005737 |
|  | 0005829 |
|  | 0032839 |
|  | 0005634 |
|  | 0043204 |
|  | 0016301 |
|  | 0000166 |
|  | 0016740 |
|  | 0000165 |
|  | 0016310 |
|  | 0043065 |
|  | 0001934 |
|  | 0006468 |
|  | 0004702 |
|  | 0035897 |
|  | 0007346 |
|  | 0002755 |
|  | 0002756 |
|  | 0035666 |
|  | 0045087 |
|  | 0009405 |
|  | 0051403 |
|  | 0034166 |
|  | 0034134 |
|  | 0034138 |
|  | 0034142 |
|  | 0034146 |
|  | 0034162 |
|  | 0038123 |
|  | 0038124 |
|  | 0002224 |
|  | 0005622 |
|  | 0016772 |
|  | 0006950 |
|  | 0008063 |
|  | 0034130 |
|  | 0033267 |
|  | 0008152 |
|  | 0005515 |

---

|  |  |
| --- | --- |
| KEGG ID | hsa:6416 |
|  | mmu:26398 |
|  | rno:287398 |

---

|  |  |
| --- | --- |
| Organism | Homo sapiens {Organism urn:agi-taxid:9606} |
|  | Mus musculus {Organism urn:agi-taxid:10090} |
|  | Rattus norvegicus {Organism urn:agi-taxid:10116} |
|  | Homo sapiens |
|  | Mus musculus |
|  | Rattus norvegicus |

---

|  |  |
| --- | --- |
| Mouse chromosome position | 11 40.53 cM |
|  | 11 33.0 cM |

---

|  |  |
| --- | --- |
| OMIM ID | 601335 |

---

|  |  |
| --- | --- |
| Rat chromosome position | 10q24 |

---

|  |  |
| --- | --- |
| Hugo ID | 6844 |
|  | HGNC:6844 |

---

|  |  |
| --- | --- |
| Human chromosome position | 17p12 |
|  | 17p11.2 |

---

|  |  |
| --- | --- |
| Swiss-Prot Accession | P45985 |
|  | P45985.1 |
|  | Q543X6 |
|  | P47809 |
|  | Q8K2U0 |
|  | P47809.2 |
|  | Q4KSH6 |
|  | B2R7N7 |
|  | B3KYB2 |
|  | D3DTS5 |
|  | Q5U0B8 |
|  | Q6FHX4 |
|  | Q6P9H2 |
|  | Q6PIE6 |
|  | B7ZA62 |

---

|  |  |
| --- | --- |
| PIR ID | I38901 |
|  | S52423 |

---

|  |  |
| --- | --- |
| GenBank ID | NC\_000017 |
|  | XR\_934080 |
|  | XR\_001752584 |
|  | XM\_005256755 |
|  | XP\_005256812 |
|  | XM\_011523977 |
|  | XP\_011522279 |
|  | XM\_011523976 |
|  | XP\_011522278 |
|  | NM\_001281435 |
|  | NP\_001268364 |
|  | NM\_003010 |
|  | NP\_003001 |
|  | XM\_005256753 |
|  | XP\_005256810 |
|  | NG\_033952 |
|  | AC005244 |
|  | AC005410 |
|  | AH006187 |
|  | AAC24130 |
|  | CH471108 |
|  | EAW89973 |
|  | EAW89974 |
|  | EAW89975 |
|  | EAW89976 |
|  | DQ015703 |
|  | AAY22176 |
|  | AA293365 |
|  | AK122997 |
|  | AK131544 |
|  | BAG54774 |
|  | AK294923 |
|  | BAG58005 |
|  | AK313053 |
|  | BAG35884 |
|  | AK316100 |
|  | BAH14471 |
|  | AK316177 |
|  | BAH14548 |
|  | AU150695 |
|  | BC036032 |
|  | AAH36032 |
|  | BC050386 |
|  | BC060764 |
|  | AAH60764 |
|  | BQ028457 |
|  | BT019676 |
|  | AAV38482 |
|  | CR536564 |
|  | CAG38801 |
|  | L31902 |
|  | L36870 |
|  | AAC41719 |
|  | U17743 |
|  | AAC50127 |
|  | P45985 |
|  | NC\_000077 |
|  | NM\_001316369 |
|  | NP\_001303298 |
|  | NM\_009157 |
|  | NP\_033183 |
|  | NM\_001316368 |
|  | NP\_001303297 |
|  | NM\_001316367 |
|  | NP\_001303296 |
|  | XM\_006533370 |
|  | XP\_006533433 |
|  | XM\_017314561 |
|  | XP\_017170050 |
|  | XM\_011249033 |
|  | XP\_011247335 |
|  | NM\_001362739 |
|  | NM\_001362740 |
|  | AL662895 |
|  | AL663069 |
|  | CH466601 |
|  | EDL10407 |
|  | AK036928 |
|  | BAC29642 |
|  | AK044534 |
|  | BAC31968 |
|  | AK044698 |
|  | BAC32038 |
|  | AK139777 |
|  | AK187893 |
|  | BC029833 |
|  | AAH29833 |
|  | BY244326 |
|  | BY277234 |
|  | CB723868 |
|  | U18310 |
|  | AAB81554 |
|  | P47809 |
|  | NC\_005109 |
|  | NM\_001030023 |
|  | NP\_001025194 |
|  | AC\_000078 |
|  | AABR07029803 |
|  | AABR07029804 |
|  | AABR07029805 |
|  | AABR07029806 |
|  | AAHX01064427 |
|  | AAHX01064428 |
|  | AAHX01064429 |
|  | CH473948 |
|  | EDM04764 |
|  | AY880882 |
|  | AAX61181 |
|  | KC795688 |
|  | AGO65350 |
|  | NC\_018928 |
|  | AF070080 |
|  | AF070081 |
|  | AF070082 |
|  | AF070083 |
|  | AF070084 |
|  | AF070085 |
|  | AF070086 |
|  | AF070087 |
|  | AF070088 |
|  | AF070089 |
|  | AF070090 |
|  | AMYH02032997 |
|  | XM\_006533369 |
|  | XP\_006533432 |
|  | XM\_006533373 |
|  | XP\_006533436 |
|  | XM\_006533372 |
|  | XP\_006533435 |
|  | AC\_000033 |
|  | AAHY01093964 |
|  | AAHY01093965 |
|  | AAHY01093966 |
|  | AAHY01093967 |
|  | AAHY01093968 |
|  | XM\_006721568 |
|  | XP\_006721631 |
|  | AC\_000149 |
|  | ABBA01054999 |
|  | ABBA01055000 |
|  | XM\_006533374 |
|  | XP\_006533437 |
|  | XM\_006533371 |
|  | XP\_006533434 |
|  | XM\_005256754 |
|  | XP\_005256811 |
|  | AABR06064274 |
|  | AABR06064275 |
|  | AABR06064276 |
|  | AABR06064277 |
|  | AABR06064278 |
|  | CAM21549 |
|  | CAM21550 |
|  | CAM23607 |
|  | CAM23608 |
|  | NT\_010718 |
|  | NW\_926584 |
|  | NW\_001838403 |
|  | AC\_000060 |
|  | CR600508 |
|  | CR601866 |
|  | NT\_096135 |
|  | NW\_001030477 |
|  | Q543X6 |
|  | Q8K2U0 |
|  | NW\_001084656 |
|  | NW\_047334 |
|  | Q4KSH6 |
|  | Q6FHX4 |
|  | Q6P9H2 |
|  | Q6PIE6 |

---

|  |  |
| --- | --- |
| Swiss-Prot ID | MP2K4\_HUMAN |
|  | MP2K4\_MOUSE |
|  | Q543X6\_MOUSE |
|  | Q4KSH6\_RAT |
|  | D3DTS5\_HUMAN |

---

|  |  |
| --- | --- |
| Cell Localization | Cytoplasm |
|  | Nucleus |

---

|  |  |
| --- | --- |
| Ensembl ID | ENSG00000065559 |
|  | ENSP00000410402.3 |
|  | ENST00000415385.7 |
|  | ENSP00000262445.5 |
|  | ENST00000353533.10 |
|  | ENSMUSG00000033352 |
|  | ENSMUSP00000041282.3 |
|  | ENSMUST00000046963.9 |
|  | ENSRNOG00000003834 |
|  | ENSRNOP00000005293.6 |
|  | ENSRNOT00000005293.7 |
|  | ENSP00000410402 |
|  | ENST00000415385 |
|  | ENSP00000262445 |
|  | ENST00000353533 |
|  | ENSMUSP00000041282 |
|  | ENSMUST00000046963 |
|  | ENSRNOP00000005293 |
|  | ENSRNOT00000005293 |

---

|  |  |
| --- | --- |
| MGI ID | MGI:1346869 |
|  | 1346869 |

---

|  |  |
| --- | --- |
| RGD ID | 1307040 |

---

|  |  |
| --- | --- |
| Unigene ID | Hs.514681 |
|  | Mm.412922 |
|  | Rn.198875 |

---

|  |  |
| --- | --- |
| Homologene ID | 48159 |

---

|  |  |
| --- | --- |
| Shape | Sickle-vertex |

---

|  |  |
| --- | --- |
| IPI ID | IPI00024674 |
|  | IPI00942214 |
|  | IPI00112335 |
|  | IPI00369884 |
|  | IPI00794157 |
|  | IPI00875050 |
|  | IPI00649311 |

---

|  |  |
| --- | --- |
| Ensembl Gene ID | ENSG00000065559 |

---

|  |  |
| --- | --- |
| ProteinAtlas NormalTissue | Tissue: cerebral cortex; Cell type: glial cells; Level: Negative; Expression type: Staining; Reliability: Uncertain |
|  | Tissue: cervix |
|  | uterine; Cell type: glandular cells; Level: Weak; Expression type: Staining; Reliability: Uncertain |
|  | Tissue: thyroid gland; Cell type: glandular cells; Level: Moderate; Expression type: Staining; Reliability: Uncertain |
|  | Tissue: vulva/anal skin; Cell type: epidermal cells; Level: Weak; Expression type: Staining; Reliability: Uncertain |
|  | Tissue: salivary gland; Cell type: glandular cells; Level: Negative; Expression type: Staining; Reliability: Uncertain |
|  | Tissue: appendix; Cell type: glandular cells; Level: Negative; Expression type: Staining; Reliability: Uncertain |
|  | Tissue: testis; Cell type: Leydig cells; Level: Weak; Expression type: Staining; Reliability: Uncertain |
|  | Tissue: nasopharynx; Cell type: respiratory epithelial cells; Level: Weak; Expression type: Staining; Reliability: Uncertain |
|  | Tissue: rectum; Cell type: glandular cells; Level: Weak; Expression type: Staining; Reliability: Uncertain |

---
